# Salivary testosterone and cortisol levels in borderline personality disorder before and after a 12-week group dialectical behavior therapy intervention

**DOI:** 10.3389/fpsyg.2023.1195187

**Published:** 2023-07-17

**Authors:** Tori Dyson, Susan J. Thomas, Michelle L. Townsend, Adam Finch, Alexandra South, Emma Barkus, Emma Walter, Carley Mendonca, Brin F. S. Grenyer, Judy A. Pickard

**Affiliations:** ^1^School of Psychology, University of Wollongong, Wollongong, NSW, Australia; ^2^School of Medicine, University of Wollongong, Wollongong, NSW, Australia; ^3^Department of Psychology, Northumbria University, Newcastle upon Tyne, North East England, United Kingdom; ^4^School of Psychology, Western Sydney University, Penrith, NSW, Australia; ^5^School of Psychology, University of Technology Sydney, Sydney, NSW, Australia

**Keywords:** borderline personality disorder, salivary cortisol, salivary testosterone, hypothalamic-pituitary-adrenal axis, hypothalamic-pituitary-gonadal axis

## Abstract

**Background:**

Borderline Personality Disorder (BPD) is a chronic, debilitating, and difficult to treat condition. BPD has recently been linked to steroid hormone dysregulation and medical conditions characterized by disturbed androgen metabolism. This study aimed to investigate cortisol and testosterone levels in BPD, and changes in hormones following psychological treatment.

**Methods:**

Participants with BPD (*n* = 33) completed a 12-week Dialectical Behavior Therapy group program. Pre and post salivary testosterone and cortisol were analyzed. Baseline hormones in the BPD group were compared to age-and-sex matched controls (*n* = 33). Non-parametric tests were utilized to investigate group differences, pre-post treatment hormone and symptom changes, and associations between symptoms and hormone levels.

**Results:**

Participants with BPD had significantly higher testosterone levels than controls. Mean testosterone levels in females with BPD were double that of female controls. Testosterone and cortisol levels were related, and some BPD symptoms were associated with with hormone levels. BPD symptoms reduced significantly with treatment, however pre to post hormone levels did not change.

**Conclusions:**

This study supports an association between BPD symptoms and neuroendocrine dysfunction at baseline, however we found no reduction in hormone dysfunction post treatment. Further research into relationships between stress signaling and neuroendocrine disturbances in BPD may inform aetiological and treatment models.

**Trial registration:**

Australian New Zealand Clinical Trials Registry ACTRN12618000477224. Registered on 3 April 2018.

## 1. Introduction

Borderline personality disorder (BPD) is characterized by symptoms of emotion dysregulation, self-injurious behaviors, and interpersonal difficulties (American Psychiatric Association, 2013). The development of BPD is currently understood to be a complex interplay of biological (genetics, temperament), psychological (comorbid anxiety, low self-esteem), and socio-environmental factors (e.g., trauma, bullying, caregiver attachment) (Stepp et al., 2016; Winsper et al., 2016; Winsper, 2018). The Biosocial Developmental Model of BPD estimates that development of this disorder begins at an epigenetic level, and builds throughout the course of infancy through to emerging traits in late adolescence (Crowell et al., 2009). The model asserts that a mismatch between infant temperament and parental response to high emotional needs represents a major risk factor, with potential to carry forward into social deficits and emotional dysregulation during childhood. Recent evidence supports positive genetic associations between BPD and Neuroticism and Openess personality traits (Streit et al., 2022). Additionally, a systematic review by Skabeikyte (2021) identified difficult childhood temperament as a risk factor for an increasing trajectory of BPD features during adolescence. These links are interpreted to indicate that both genetic (personality and temperament) and environmental factors (e.g., early life interpersonal traumatisation) may contribute to the development of BPD (Streit et al., 2022). These factors together may contribute to maladaptive traits in cognitive, interpersonal, emotional, and behavioral domains that develop in adolescence, and often continue through to adulthood unless early intervention is provided (Winsper et al., 2016; Winsper, 2018).

Personality disorders are estimated to impact approximately 7.8 % of the population worldwide, with a prevalence of 2.8% for cluster b (antisocial, borderline, narcissistic and histrionic) disorders (Winsper et al., 2020). Despite its prevalence, BPD continues to be difficult to treat and many affected people experience symptoms across their lifespan (Cristea et al., 2017). Psychotherapy is currently identified as a first line intervention for BPD, with pharmacotherapy (e.g., antidepressants, mood stabilizers, and antipsychotics) recommended as a secondary or concurrent intervention (National Institute for Health and Clinical Excellence (NICE), 2011). However, pharmacotherapy does not change the course of the disorder and is predominantly used to treat co-morbid conditions and specific symptoms (e.g., depression, anxiety, mood lability) (National Collaborating Centre for Mental Health (UK), 2009).

While evidence-based treatments are available, these are relatively labor-intensive, with high attrition rates (Landes and Chalker, 2016). Some individuals do not seek or respond to treatment (Woodbridge et al., 2021) and continue to experience symptoms long-term. The higher rates of suicidality, risk taking behavior, and self-harm within this population represents significant personal, psychological, social and health care costs (Cristea et al., 2017). Given the limitations of pharmacological and psychological treatments for BPD, there is a need for targeted interventions.

Emerging research is examining the potential role of neuroendocrine dysfunction in the development and maintenance of symptoms of BPD, which may inform aetiological models and intervention approaches. Elevated impulsivity, aggression, risk taking, emotion dysregulation, and interpersonal difficulties have been linked to atypical cortisol and testosterone levels in healthy and clinical populations (Rausch et al., 2015; Winsper et al., 2016), suggesting potential relevance to BPD symptoms. Cortisol is a commonly studied biomarker of stress and functioning of the hypothalamic-pituitary-adrenal (HPA) axis, which regulates stress signaling and stimulates the release of adrenocortico-trophic hormone (ACTH) from the pituitary. ACTH, in turn, stimulates adrenal cortisol release. Previous studies have reported higher basal cortisol levels within BPD samples (Lieb et al., 2004; Rausch et al., 2015). Other studies have found no such effect, or only in BPD participants with co-morbid trauma histories (Jogems-Kosterman et al., 2007; Dettenborn et al., 2016). A recent review and meta-analysis by Thomas et al. (2019) reported inconsistencies within the cortisol and BPD literature, with some studies reporting higher and some lower cortisol levels in individuals with BPD, however only 12 studies were available. This highlights the need for further research to better understand the complex nature of HPA-axis dysfunction in individuals with BPD. The HPA axis is known to share close bidirectional influences with the hypothalamic-pituitary-gonadal (HPG) axis to support the balance of reproduction and survival (Acevedo-Rodriguez et al., 2018). Testosterone, the main male sex hormone, is an output of the HPG axis and is synthesized in the testes and in smaller quantities in the ovaries (Sher, 2013). Although males produce around ten times the testosterone of females, it plays important physiological roles and is linked to cognitive functioning and psychological wellbeing in both sexes, with females being more sensitive to its effects (Sher, 2013). Bidirectional influences between the HPA and HPG axes are indicated through the effects of stress on reproductive functions and the effects of sex hormones on psychological wellbeing (Liening et al., 2010; Zilioli et al., 2015). Previous studies (Zilioli et al., 2015) provide support for the dual-hormone hypothesis positing that cortisol and testosterone levels are often related due to the modulating role of the HPA and HPG axes on each another. Higher levels of testosterone have been shown to influence aggression and risk taking behavior (Montoya et al., 2012; Mehta et al., 2015). Interestingly, a high rate of comorbidity of polycystic ovary syndrome (PCOS), a condition characterized by increased testosterone, has been noted in females with BPD (Roepke et al., 2010; Rausch et al., 2015; Dettenborn et al., 2016; Trisno et al., 2016; Tan et al., 2018). As well as being associated with reproductive, endocrine and metabolic features, PCOS is linked to psychological distress and negative mood states including anger and hostility (Borghi et al., 2018). These findings lead to the suggestion that testosterone disturbances may be associated with symptoms of BPD. There is currently a lack of research reporting correlations between BPD symptoms and testosterone levels however higher testosterone levels in BPD have been proposed as a potential mechanism contributing to aggressive symptoms in BPD (Rausch et al., 2015; Dettenborn et al., 2016).

It is plausible that understanding neuroendocrine dysfunction could lead to improved understanding of BPD etiology. Studies have begun to explore changes in HPA axis activity in response to pharmacological treatments, but not psychological treatments (Rinne et al., 2003). Furthermore, while hormone level changes over time in response to psychological treatment has been assessed in other clinical conditions such as PTSD (Olff et al., 2007), to our knowledge there are no studies of hormone changes in BPD associated with psychological treatment. Therefore, a greater understanding of neuroendocrine changes in response to psychological treatments may inform more specific interventions to support those experiencing this complex mental health issue.

The aim of this study was to measure baseline levels of cortisol and testosterone in participants diagnosed with BPD compared with matched controls. We also aimed to measure hormone levels in relation to a 12-week group manualised DBT-based group intervention, by comparing pre-post hormone levels and relationships to symptom levels and symptom changes. We hypothesize that: (1) Participants with BPD will have higher levels of testosterone compared with matched controls, (2) Testosterone and cortisol levels in participants with BPD will be positively related to BPD symptoms (3) There will be an association between testosterone and cortisol levels (3) There will be a significant reduction in testosterone and cortisol levels in particpants with BPD following treatment, and (4) Hormone changes will be associated with symptom change following treatment.

## 2. Method

### 2.1. Participants

Due to missing data the final sample comprised of 33 participants in the BPD group (*n* = 28, 84.8% female) between the ages of 18 and 59. The BPD group were participating in a longitudinal study examining the efficacy of a stepped care treatment model (Pickard et al., 2021) and were invited to participate in a sub-study investigating hormone levels. Recruitment for the BPD group occurred via the local community mental health central intake line. Referrals that appeared to endorse symptoms indicative of BPD (e.g., emotional dysregulation, self-harm, and suicidal behaviors) were invited to undergo the Structured Clinical Interview for DSM-5 (SCID-5) to verify a BPD diagnosis conducted by a trained psychologist. Participants provided their written consent and were randomized to the treatment condition, where they were offered a 12-week group treatment at the local community mental health service (which was step 1 of the larger stepped care treatment program), or continued their treatment as usual (TAU). The full inclusion and exclusion criteria and randomization protocol of the original study are published elsewhere (Pickard et al., 2021). The present study utilized questionnaire data collected during the group treatment, for those participants who also consented to participate in the saliva sub-study. Baseline hormone levels were then compared to age and sex matched healthy controls (*n* = 33) analyzed using the same methods. The study was approved by the University of Wollongong's Human Ethics Committee (HREC no. 2017/362). Exclusion criteria for the control group were neurological conditions, sleep disorders, kidney or liver disease, current treatment for diabetes, taking psychoactive medications, St John's Wort or steroids.

### 2.2. Procedures

#### 2.2.1. Group treatment

the BPD group engaged in weekly treatment sessions over a 12-week period at the local mental health service by two trained psychologists. treatment provided was manualised according to dialectical behavior therapy (DBT) principles, which is an evidenced based treatment for BPD that provides skills in mindfulness, emotional regulation, distress tolerance, and interpersonal effectiveness (Linehan, 1987). sessions were 2 h each in duration.

#### 2.2.2. Clinical evaluation

Participants with BPD were clinically assessed pre and post treatment as previously reported (Pickard et al., 2021) and specific measures in this study are as follows.

The Inventory of Statements About Self-injury (ISAS) is a well validated self-report measure examining the frequency and function of non-suicidal self-injury, and has shown sensitivity and consistency within a BPD population (Klonsky and Glenn, 2009). The “functions” section of this measure comprised of 39 items with 3 response options “0 – not relevant to me”, “1 – somewhat relevant to me”, and “2 – very relevant to me”. This measure was selected for use as an outcome measure relating to aggression toward the self and impulsivity. From item scores, 13 subscales are derived: (1) affect regulation, (2) anti-dissociation, (3) anti-suicide, (4) marking distress, (5) self-punishment, (6) autonomy, (7) interpersonal boundaries, (8) interpersonal influence, (9) peer bonding, (10) revenge, (11) self-care, (12) sensation seeking, and (13) toughness. Detailed explanations of these subscales are described elsewhere (Klonsky and Glenn, 2009).

Participants were asked to rate each of the DSM-5 BPD criteria problem areas for its current severity over the past 2 weeks (1 = *none of the time* to 6 = *all of the time*) in order to measure BPD symptomology, an approach that has previously demonstrated good internal consistency and predictive validity (Miller et al., 2018). Reliability for the measure of BPD symptoms has been previously reported α = 0.819 (Woodbridge et al., 2021). We asked two questions in relation to DSM-5 BPD Criterion 9; a question about distrust (paranoia), and a question about things feeling unreal (dissociative experiences), following the McLean Screening Instrument for BPD (MSI-BPD) method (Zanarini et al., 2003). An MSI-BPD cut off score of 7 was used to indicate current caseness.

Finally, the Difficulties in Emotion Regulation Scale (DERS) is a measure designed to capture multiple dimensions of emotion regulation and dysregulation (Gratz and Roemer, 2004). The DERS has also demonstrated clinical utility within a BPD sample receiving outpatient DBT group treatment, which is a similar sample to the current study (Osborne et al., 2017; Hallion et al., 2018). The DERS is comprised of 41 items and items are rated according to relevance to the responder. Responses are “1 – almost never”, “2 – sometimes”, “3 – about half the time”, “4 – most of the time”, and “5 – almost always”. This measure was selected as an outcome measure for treatment. Higher scores on this measure also indicate greater dysfunction in emotional regulation, and five subscales are procured from item score patterns: (1) non-acceptance of emotional responses, (2) difficulty engaging in goal-directed behavior, (3) impulse control difficulties, (4) lack of emotional awareness, (5) limited access to emotion regulation strategies, and (6) lack of emotional clarity. Detailed descriptions of each subscale are described elsewhere (Hallion et al., 2018).

### 2.3. Saliva sampling

BPD group saliva samples were taken between 10 and 11 AM in week one and week twelve of group treatment and stored frozen at −30°C until assay. All samples underwent one freeze thaw cycle prior to analyses. The matched control group data was derived from an existing pool of data that were sampled between 2 and 4 PM. Due to the different time of sampling between groups, the between-group comparisons for cortisol are not interpreted, as these may be affected by diurnal cortisol variations.

#### 2.3.1. Assay procedure

On the day of assay, samples were thawed and analyzed using commercially available kits (Salimetrics, USA) according to the manufacturer's instructions. Thawed samples were centrifuged at 1500 × g for 15 min to collect clear saliva and this saliva was used without further processing for all assays. All samples were brought to room temperature before adding to the assay wells and all samples were analyzed in duplicate. The cortisol assay had a sensitivity of 0.003 μg/dL, with an intra assay variability of 4%, and an inter assay variability of 5.4%. The testosterone assay had a sensitivity of 1 pg/dL with an intra assay variability of 3.2%, and inter assay variability of 4.6%.

### 2.4. Statistical analyses

Data processing was performed using SPSS version 27.0.1.0 (SPSS, IBM, USA).

Independent-Samples Mann–Whitney *U*-tests were utilized to test our initial hypothesis that testosterone and cortisol level differences would be observed between the BPD group (*n* = 33, 28 female) and matched controls (*n* = 33, 28 female). We then utilized the pre and post treatment data (repeated measures) in the BPD group only, to test the remaining hypotheses. Males were excluded from pre-post analyses due to sex hormone differences, coupled with the disproportionately small male proportion (males comprised 15.2% of the sample, *n* = 5). The onset of the global SARS-2-CoV pandemic prevented further saliva collection, this combined with natural attrition in previous groups resulted in a reduced sample size, *n* = 17 female participants remained with pre-and-post treatment data utilized for analyses. *T*-tests were conducted to determine if there was a significant difference in baseline hormone profiles between participants who completed all measures across time and those that only completed measures at Time 1. No significant difference was found.

We then investigated if hormone levels and BPD symptom levels changed between pre-post group treatment, using Wilcoxon signed ranks tests. To investigate relationships between testosterone and cortisol levels, of relevance to the dual hormone hypothesis, we correlated cortisol and testosterone levels and symptoms in the pre and post treatment conditions. Pre and post hormone and psychometric measure change scores were then calculated to investigate whether hormone change was associated with symptom change, using Spearman's correlations. It was hypothesized that changes in hormone levels from pre to post treatment would be associated with reductions in BPD symptomology as measured by psychometric data.

## 3. Results

### 3.1. Participant characteristics

Participant characteristics, hormone levels and psychometric scores are provided in Table 1.

**Table 1:** Participant characterisitcs, hormone levels, and psychometric scores, by group.

**Characteristic**	**Matched controls (baseline only)**		**BPD group Pre-treatment (baseline)**		**BPD group Post-treatment**
	**M (*n =* 5)**	**F (*n =* 28)**	**M (*n =* 5)**	**F (*n =* 28)**	**M (excluded) F (*n =* 17)**
**Age** (years)	35.8 (10.43)	28.32 (9.47)	36.80 (9.15)	28.36 (9.6)	28.65 (10.48)
**Cortisol** (μg/ml)	0.118 (0.032)	0.135 (0.084)	0.156 (0.094)	0.235 (0.147)	0.262 (0.173)
**Testosterone** (pg/ml)	177.700 (40.832)	55.252 (18.071)	236.450 (168.690)	111.580 (56.786) (*n =* 17)	117.415 (69.191)
**DERS subscales**					
Nonacceptance				23.19 (5.37)	21.30 (5.15)
Goals				19.85 (3.91)	19.55 (4.77)
Impulsivity				23.50 (5.16)	20.45 (5.44)
Awareness				19.70 (4.84)	17.10 (4.68)
Strategies				30.63 (6.63)	27.90 (7.13)
Clarity				17.07 (3.68)	15.70 (3.08)
**ISAS subscales**					
Total				27.73 (14.58)	24.75 (13.70)
Affect regulation				3.92 (2.23)	4.10 (1.92)
Interper. Boundaries				1.38 (1.55)	1.20 (1.54)
Self-punishment				4.27 (2.24)	3.65 (2.16)
Self-care				2.08 (1.94)	1.65 (1.95)
Anti-dissociation				3.23 (2.18)	3.06 (2.09)
Anti-suicide				3.42 (2.19)	3.00 (2.18)
Sensation-seeking				1.15 (1.49)	0.85 (1.46)
Peer-bonding				0.50 (1.21)	0.35 (1.09)
Interper. influence				1.31 (1.26)	1.30 (1.30)
Toughness				1.62 (1.90)	1.20 (1.44)
Distress				2.96 (1.95)	3.10 (1.97)
Revenge Autonomy				0.96 (1.71)	0.85 (1.69)
				0.92 (1.55)	0.47 (1.02)
***BPD Symptom severity***					
Unstable relationships				3.29 (1.57)	2.59 (1.73)
Self-harm				1.94 (2.82)	1.5 (2.00)
Impulsivity				3.41 (1.22)	2.76 (1.25)
Anger				3.00 (1.37)	2.47 (1.23
Real or imagined abadonement				2.76 (1.72)	2.41 (1.91)
Identity disturbances				2.59 (1.42)	2.47 (1.38)
Mood dysregulation				3.60 (1.11)	2.69 (1.45)
Chronic emptiness				3.35 (1.12)	3.00 (1.58)
Paranoid Ideation				2.88 (1.65)	2.27 (1.83)
**MSI-BPD (original measure)**					
Total				26.48 (6.66)	21.97 (9.10)

NB: values are represented as mean (SD). Hormone levels are reported to three decimal places.

### 3.2. Group comparison of hormone differences at baseline

Independent-Samples Mann–Whitney *U*-tests were conducted to determine if there were differences in hormone levels (testosterone and cortisol) between the matched controls and BPD group. Cortisol scores for BPD participants (mean rank = 40.14) were significantly higher than for controls (mean rank = 26.86), *U* = 763.50, *z* = 2.809, *p* = 0.005. Testosterone scores for BPD participants (mean rank = 42.27) were also significantly higher than for controls (mean rank = 24.73), *U* = 834.000, *z* = 3.713, *p* = < 0.001.

### 3.3. Hormone related change in BPD group across treatment

Wilcoxon signed rank tests were used to investigate changes in cortisol and testosterone levels from pre (baseline) to post-treatment (*n* = 17). Cortisol elicited a non-significant median change between pre and post-group treatment, *z* = −1.823, *p* 0.068, two tailed. Relative to their pre-treatment cortisol levels, 5 participants had reduced cortisol levels (sum of ranks = 115), whilst 12 participants had increases in cortisol levels. No ties were observed.

Testosterone also showed a non-significant median change at *z* = −0.308, *p* = 0.758, two tailed. Relative to their pre-treatment testosterone levels 7 participants experienced a reduction in the amount of testosterone (sum of ranks = 83), whilst 10 participants experienced an increase in testosterone. No ties were observed.

### 3.4. Symptom level change in BPD group across treatment

A Wilcoxon signed rank test was used to examine changes in the number of BPD symptoms endorsed on the McLean's Screening Measure for Borderline Personality Disorder (MSI-BPD) from pre (baseline) to post-treatment (*n* = 17). BPD symptom scores reduced between pre-treatment and post-treatment reaching statistical significance at, *z* = −2.142 (corrected for ties), ties = 7, *p* = 0.03, two tailed. Relative to their pre-treatment rankings, 8 participants experienced a reduction in the amount of endorsed BPD symptoms on the MSI-BPD (sum of ranks = 48), whilst 2 participants experienced an increase in symptoms, and 7 reported the same amount of symptoms post-treatment. A *large* effect size was observed, at *r* = 0.52.

To determine change in severity of BPD symptoms a Wilcoxon signed rank test was conducted on total severity scores on the McLean's Screening Measure for Borderline Personality Disorder (MSI-BPD) from pre to post treatment. Pre treatment scores (*Mdn* = 27.60) showed a significant decrease compared with Post treatment scores (*Mdn* = 20.00), *T* = 24.5, *p* = 0.024. Relative to their pre-treatment rankings, 11 participants reported a reduction in the severity of endorsed BPD symptoms on the MSI-BPD, whilst 5 participants experienced an increase in symptom severity, and 1 participant reported the same severity of symptoms post-treatment. A small effect size was observed, at *r* = 0.39.

Wilcoxon signed rank tests were then conducted on individual symptoms to determine the effectiveness of treatment on specific BPD symptoms. A significant change in severity for mood symptoms from pre treatment (*Mdn* = 4.00) with post treatment scores (*Mdn* = 2.00), *T* = 0.45, *p* = 0.007. A reduction in mood symptoms was reported by eight participants post treatment, with seven participants reporting no change in mood symptoms. A large effect size was observed at *r* = 0.69. A non-significant decrease in remaining symptoms was noted from pre treatment to post treatment scores.

### 3.5. Correlations between hormones and BPD symptoms

Two-tailed Spearman's correlations were utilized to investigate associations between hormones and symptoms at time 1 (Table 2) for the female BPD group participants (*n* = 17). Salivary cortisol and testosterone levels showed strong positive correlations at pre-treatment (baseline) (*r* = 0.632, *p* = 0.003). The same correlations were performed post-intervention (Table 3). Post-treatment cortisol levels were moderately correlated with testosterone, impulse control difficulties, limited access to self-regulation strategies, and strongly correlated with lack of emotional clarity. Post-treatment testosterone levels were moderately correlated with total frequency and function of non-suicidal self-injury (ISAS total scores) and total BPD symptom severity (MSI-BPD total scores). The same correlations were performed on the change scores, however there were no significant correlations between hormone change and symptom change scores (not shown).

**Table 2:** Baseline Spearman's correlations for variables in female participants with borderline personality disorder.

	**1**	**2**	**3**	**4**	**5**	**6**	**7**	**8**	**9**	**10**
1. Cortisol	–									
2. Testosterone	0.632^**^	–								
3. ISAS total score	0.016	0.243	–							
**DERS subscales**										
4. Non–acceptance	0.232	0.097	0.014	–						
5. Goals	−0.052	0.009	0.115	0.585^*^	–					
6. Impulse	−0.055	−0.031	0.416	0.141	0.506^*^	–				
7. Awareness	0.192	0.095	0.326	0.112	−0.273	0.137	–			
8. Strategies	0.142	0.25	0.422	0.328	0.579^*^	0.589^*^	0.081	–		
9. Clarity	−0.045	−0.045	−0.024	0.254	0.061	0.188	0.392	−0.034	–	
10. MSI–BPD severity total score	0.173	0.058	0.350	0.148	0.172	0.358	0.232	0.297	0.439	
11. MSI–BPD symptom total score	0.430	0.377	0.037	0.097	0.005	−0.079	0.379	−0.023	−0.035	0.272

* Correlation is significant at the 0.05 level (2-tailed).

** Correlation is significant at the 0.01 level (2-tailed).

**Table 3:** Post-treatment Spearman's correlations between variables in female participants with borderline personality disorder.

	**1**	**2**	**3**	**4**	**5**	**6**	**7**	**8**	**9**	**10**
1. Cortisol	–									
2. Testosterone	0.583^*^	–								
3. ISAS total score	0.070	0.546^*^	–							
**DERS Subscales**										
4. Non-acceptance	0.432	0.374	0.587^*^	–						
5. Goals	0.311	0.279	0.440	0.616^**^	–					
6. Impulse	0.548^*^	0.407	0.509^*^	0.777^**^	0.470	–				
7. Awareness	0.358	0.252	0.196	0.381	−0.048	0.452	–			
8. Strategies	0.512^*^	0.461	0.599^*^	0.729^**^	0.799^**^	0.757^**^	0.122	–		
9. Clarity	0.663^**^	0.315	0.281	0.560^*^	0.171	0.764^**^	0.581^*^	0.529^*^	–	
10. MSI-BPD total severity score	0.283	0.405	0.538^*^	0.509^*^	0.327	0.712^**^	0.568^*^	0.649^**^	0.626^**^	
11. MSI-BPD symptom total score	0.467	0.557^*^	0.604^*^	0.420	0.453	0.699^**^	0.382	0.689^**^	0.588^*^	0.819^**^

* Correlation is significant at the 0.05 level (2-tailed).

** Correlation is significant at the 0.01 level (2-tailed).

## 4. Discussion

This study was the first, to our knowledge, to explore hormone levels in individuals with BPD over the course of DBT treatment. We also completed one of the few comparisons of hormone levels between particpants with BPD and healthy controls. Additionally, we examined relationships between cortisol and testosterone levels to better understand links between HPG and HPA axis functioning. Further, we examined correlations between cortisol and testosterone levels and BPD symptoms.

We predicted that the BPD group would have higher levels of testosterone compared with matched controls, based on the findings of prior studies investigating neuroendocrine dysfunction, and the observation of higher prevalence of medical conditions characterized by endocrine dysfunction and disturbed androgen metabolism in BPD (Rausch et al., 2015; Dettenborn et al., 2016; Tan et al., 2018). This prediction was supported, with higher salivary levels of testosterone in the BPD group compared with matched controls, suggesting neuroendocrine involvement in BPD. Our finding of increased testosterone in females with BPD is consistent with prior findings (Roepke et al., 2010; Rausch et al., 2015; Dettenborn et al., 2016). This study therefore adds to a small but growing body of literature indicating that BPD is associated with neuroendocrine dysfunction, particularly disturbed androgen metabolism.

We also predicted that cortisol and testosterone levels would be related, in view of the dual-hormone hypothesis. The hypothesis asserts that the HPA and HPG axes modulate one another, which is supported by prior studies of healthy and clinical samples (Zilioli et al., 2015). In the current study, baseline testosterone and cortisol results were strongly postively correlated across the BPD and control participants, supporting hypothesized relationships between the HPA and HPG axes. It may be that relationships between cortisol, testosterone levels and BPD in the current and previous research are linked to stress exposure and development of maladaptive stress responses. Childhood adversity is a strong risk factor for BPD (Ferber et al., 2021) and repeated or chronic activation of the HPA axis may lead to disturbances in stress reactivity. Due to the crossover between the HPA and HPG axes, chronic activation of the HPA axis may lead to HPG dysregulation, androgen disturbances and increased risk of metabolic conditions (Ferber et al., 2021).

We predicted reductions in cortisol and testosterone levels between pre and post treatment in the BPD group. Our results indicated that there were no significant differences in hormone levels between the two time points. Therefore, in the present sample, hormone levels did not significantly change in response to treatment. However, many participants significantly improved following the group DBT treatment, having a reduction in BPD symptoms endorsed on the McLean's Screening Instrument for Borderline Personality Disorder (MSI-BPD). Therefore, treatment may provide psychological means to increase coping and reduce symptoms, which may not directly be reflected in changes to hormone levels in the time period of the study. BPD is a complex disorder that can have severe impact upon intrapersonal and interpersonal functioning and has been related to genetic vulnerability. The group treatment included in the current study is relatively brief for the complexity and severity of dysfuntion associated with BPD. While there was a significant reduction in symptoms endorsed by participants, it is plausible that the non-significant change in hormone levels may be associated with dose level, i.e., 12 sessions or time required for change in hormone levels (>12 weeks). Given BPD is established by adolescence and relationships between stress and altered hormonal functioning of the HPA and HPG axes are likely to be chronic, further prospective research over longer time periods would aid in understanding associations between BPD, steroid and androgen hormone dysregulation and associated medical conditions.

Importantly we found correlations between the number of BPD symptoms endorsed BPD symptom-severity and both cortisol and testosterone. Post-treatment cortisol levels were moderately correlated with impulse control difficulties, limited access to self-regulation strategies, and strongly correlated with lack of emotional clarity. Post-treatment testosterone levels were moderately correlated with total frequency and function of non-suicidal self-injury and total BPD symptom severity. This study expands on previous findings of higher testosterone levels in BPD (Rausch et al., 2015; Dettenborn et al., 2016) by showing direct correlations between BPD symptoms and testosterone levels. Links between BPD symptoms and hormone levels may have important implications for aetiological models and intervention approaches and the current findings therefore warrant further investigation. In particular, it would be of interest to further investigate links between neuroendocrine dysregulation, specific symptom types and higher level neurobiological functions such as impaired inhibition.

Additionally, we also predicted that hormone related change would be associated with symptom change following treatement. The concept that hormone dysfunction could be influenced by psychological treatment has been explored in other clinical populations (e.g., in PTSD, see Olff et al., 2007). However, this is the first study to our knowledge in individuals with BPD. We did not find significant associations between these variables, however, more research is needed with larger samples. Current evidence supports several psychological interventions such as, schema therapy, psychodynamic and mentalisation based approaches as efficacious in treating BPD symptoms (Katakis et al., 2023). It is plausible these alternative treatments related to both length and theoretical orientation may expect similar findings.

The current study had several strengths. Firstly, the utilization of a structured clinical measure (SCID-V) to verify BPD diagnosis in participants, and recruitment through a local community mental health service likely increased diagnostic validity, as prior studies have relied on self-reporting of symptoms for inclusion (Cristea et al., 2017). Secondly, to our knowledge this is the first longitudinal study of BPD investigating hormone level changes over the course of psychological treatment and associations to self report measures. Additionally, the inclusion of both testosterone and cortisol allowed consideration of HPA and HPG axis involvement and associations.

Some methodological limitations need to be discussed. Firstly, the sample size was relatively small. Previous studies of testosterone in BPD have had samples sizes of *n* = 18, *n* = 55 and *n* = 30 participants with BPD (Roepke et al., 2010; Rausch et al., 2015; Dettenborn et al., 2016), highlighting the paucity of research and need for further studies with larger sample sizes. Additionally, the BPD participants reported a range of medications and medical conditions, whereas the controls did not. Another methodological limitation was the different timing of saliva collection between groups. The BPD group had morning saliva collections, whereas control data was collected in the afternoon. Because of diurnal cortisol variations, we have not interpreted the between-group differences in cortisol levels, but focused on testosterone. Testosterone levels are relatively stable during the course of the day, and are unlikely to be impacted by differences in sampling times (Liening et al., 2010).

Another methodological limitation is the exclusion of male participants in the secondary analyses, which investigated hormone and self report measures over the course of treatment. Despite there being similar prevalences across sex for BPD, males are often underrepresented within BPD samples (Sansone and Sansone, 2011), and this was also evident in our treatment group. Due to sex differences in testosterone and cortisol levels (DeSoto and Salinas, 2015), males were included for the comparison with sex-matched controls, but removed from the pre-post treatment analyses where the control group was no longer involved.

The results indicate that the complex relationships between BPD and endocrine dysregulation warrant further research to better understand between-group differences, sex differences and relationships to symptoms and treatments. Additionally, further research is needed to understand how neuroendocrine disturbances may link to other, higher level, neurobiological processes such as impaired inhibition by the frontal cortex.

## 5. Conclusions

Testosterone levels in females with BPD were double that of matched controls. There were moderate-strong correlations between cortisol and testosterone and BPD symptoms in females with BPD. Therefore, our study provides further evidence of neuroendocrine dysfunction in BPD, associated with the HPG axis. Testosterone levels were also strongly correlated with cortisol levels, supporting an interplay between stress signaling and HPG activity. Further research is needed to understand relationships between stress exposure, stress signaling and related neuroendocrine disturbances. The current findings add to a small but growing number of studies finding testosterone association with BPD. In conjunction with other studies they support a need for integrative (physical and psychological) assessment and treatment guidelines for this complex mental health issue and its associated physical health conditions.

## Data availability statement

The raw data supporting the conclusions of this article will be made available by the authors, without undue reservation.

## Ethics statement

The studies involving human participants were reviewed and approved by the University of Wollongong Health Research Ethics Committee. The patients/participants provided their written informed consent to participate in this study.

## Author contributions

TD was responsible for data analysis and manuscript development. ST contributed to research design, coordination of control data, data analysis, and manuscript development. MT was responsible for data storage and manuscript review. AF was responsible for recruitment, data collection, and treatment delivery. BG contributed to review of the manuscript. AS, EB, EW, and CM were responsible for collection and access to control data. JP (CI) was responsible for research design, data analysis, and manuscript development. All authors contributed to the article and approved the submitted version.

## References

[B1] Acevedo-Rodriguez A. Kauffman A. Cherrington B. Borges C. Roepke T. A. Laconi M. (2018). Emerging insights into hypothalamic-pituitary-gonadal axis regulation and interaction with stress *signalling*. JNE 30, e12590. 10.1111/jne.12590 29524268 PMC6129417

[B2] American Psychiatric Association (2013). Diagnostic and Statistical Manual of Mental Disorders - DSM-5. Anew York, NY: American Psychiatric Publishing.

[B3] Borghi L. Leone D. Vegni E. Galiano V. Lepadatu C. Sulpizio P. Gynecology.. (2018). Psychological distress, anger and quality of life in polycystic ovary syndrome: associations with biochemical, phenotypical andsocio-demographic *factors*. J. Psych. 28, 128–137. 10.1080/0167482X.2017.1311319 28385114

[B4] Cristea I. A. Gentili C. Cotet C. D. Palomba D. Barbui C. Cuijpers P.. (2017). Efficacy of psychotherapies for borderline personality disorder: a systematic review and meta-analysis. JAMA psychiatry 74, 319–328. 10.1001/jamapsychiatry.2016.4287 28249086

[B5] Crowell S. E. Beauchaine T. P. Linehan M. M. (2009). A biosocial developmental model of borderline personality: elaborating and extending linehan's theory. Psychol. Bull. 135, 495. 10.1037/a0015616 19379027 PMC2696274

[B6] DeSoto M. C. Salinas M. (2015). Neuroticism and cortisol: the importance of checking for sex differences. Psychoneuroendocrinology 62, 174–179. 10.1016/j.psyneuen.2015.07.608 26318627

[B7] Dettenborn L. Kirschbaum C. Gao W. Spitzer C. Roepke S. Otte C.. (2016). Increased hair testosterone but unaltered hair cortisol in female patients with borderline personality disorder. Psychoneuroendocrinology 71, 176–179. 10.1016/j.psyneuen.2016.05.026 27290653

[B8] Ferber S. G. Hazani R. Shoval G. (2021). Targeting the endocannabinoid system in borderline personality disorder: corticolimbic and hypothalamic perspectives. Curr. Neuropharmacol. 19, 360–371. 10.2174/1570159X18666200429234430 32351183 PMC8033970

[B9] Gratz K. L. Roemer L. (2004). Multidimensional assessment of emotion regulation and dysregulation: development, factor structure, and initial validation of the difficulties in emotion regulation scale. J. Psychopathol. Behav. Assess. 26, 41–54. 10.1023/B:JOBA.0000007455.08539.94

[B10] Hallion L. S. Steinman S. A. Tolin D. F. Diefenbach G. J. (2018). Psychometric properties of the difficulties in emotion regulation scale (ders) and its short forms in adults with emotional disorders. Front. Psychol. 9, 539. 10.3389/fpsyg.2018.00539 29725312 PMC5917244

[B11] Jogems-Kosterman B. J. M. Knijff D. Kusters D. W. W. van Hoof R. J. J. M. (2007). Basal cortisol and DHEA levels in women with borderline personality disorder. J. Psychiatr. Res. 41, 1019–1026. 10.1016/j.jpsychires.2006.07.019 17028025

[B12] Katakis P. Schlief M. Barnett P. (2023). Effectiveness of outpatient and community treatments for people with a diagnosis of ‘personality disorder': systematic review and meta-analysis. BMC Psychiatry 23, 57. 10.1186/s12888-022-04483-0 36681805 PMC9862782

[B13] Klonsky E. D. Glenn C. R. (2009). Assessing the functions of non-suicidal self-injury: psychometric properties of the inventory of statements about self-injury (ISAS). J. Psychopathol. Behav. Assess. 31, 215–219. 10.1007/s10862-008-9107-z 29269992 PMC5736316

[B14] Landes S. J. Chalker S. A. (2016). Predicting dropout in outpatient dialectical behavior therapy with patients with borderline personality disorder receiving psychiatric disability. Borderline Pers. Disord. Emot. Dysreg. 3, 1–8. 10.1186/s40479-016-0043-3 27588205 PMC5007727

[B15] Lieb K. Rexhausen J. E. Kahl K. G. Schweiger U. Philipsen A. Hellhammer D. H.. (2004). Increased diurnal salivary cortisol in women with borderline personality disorder. J. Psychiatr. Res. 38, 559–565. 10.1016/j.jpsychires.2004.04.002 15458851

[B16] Liening S. H. Stanton S. J. Saini E. K. Schultheiss O. C. (2010). Salivary testosterone, cortisol, and progesterone: two-week stability, interhormone correlations, and effects of time of day, menstrual cycle, and oral contraceptive use on steroid hormone levels. Physiol. Behav. 99, 8–16. 10.1016/j.physbeh.2009.10.001 19833145

[B17] Linehan M. M. (1987). Dialectical behavior therapy for borderline personality disorder: theory and method. Bull. Menninger Clin. 51, 261. 3580661

[B18] Mehta P. H. Welker K. M. Zilioli S. Carré J. M. (2015). Testosterone and cortisol jointly modulate risk-taking. Psychoneuroendocrinology 56, 88–99. 10.1016/j.psyneuen.2015.02.023 25813123

[B19] Miller C. E. Lewis K. L. Huxley E. Townsend M. L. (2018). A 1-year follow-up study of capacity to love and work: what components of borderline personality disorder most impair interpersonal and vocational functioning? Pers. Mental Health, 12, 334–344. 10.1002/pmh.1432 30136443

[B20] Montoya E. R. Terburg D. Bos P. A. Van Honk J. (2012). Testosterone, cortisol, and serotonin as key regulators of social aggression: a review and theoretical perspective. Motiv. Emot. 36, 65–73. 10.1007/s11031-011-9264-3 22448079 PMC3294220

[B21] National Collaborating Centre for Mental Health (UK) (2009). Borderline Personality Disorder: Treatment and Management. London: British Psychological Society. 21796831

[B22] National Institute for Health Clinical Excellence (NICE). (2011). Common Mental Health Problems: Identification and Pathways to Care (CG123). Available online at: https://www.nice.org.uk/guidance/cg123/

[B23] Olff M. Vries D. Güzelcan G. J. Assies Y. Gersons J. B.P.R. (2007). Changes in cortisol and DHEA plasma levels after psychotherapy for PTSD. Psychoneuroendocrinology 32, 619–626. 10.1016/j.psyneuen.2007.04.001 17570603

[B24] Osborne T. L. Michonski J. Sayrs J. Welch S. S. Anderson L. K. (2017). Factor structure of the difficulties in emotion regulation scale (DERS) in adult outpatients receiving dialectical behavior therapy (DBT). J. Psychopathol. Behav. Assess. 39, 355–371. 10.1007/s10862-017-9586-x

[B25] Pickard J. A. Finch A. Huxley E. Townsend M. L. Deuchar S. Lewis K. L.. (2021). Assessing the efficacy of a stepped-care group treatment programme for borderline personality disorder: study protocol for a pragmatic trial. Trials 22, 1–9. 10.1186/s13063-021-05327-0 34099033 PMC8183092

[B26] Rausch J. Gäbel A. Nagy K. Kleindienst N. Herpertz S. C. Bertsch K.. (2015). Increased testosterone levels and cortisol awakening responses in patients with borderline personality disorder: gender and trait aggressiveness matter. Psychoneuroendocrinology 55, 116–127. 10.1016/j.psyneuen.2015.02.002 25796037

[B27] Rinne T. Kloet D. Wouters E. R. Goekoop L. de Rijk J. G. van den Brink R. H.. (2003). Fluvoxamine reduces responsiveness of HPA axis in adult female BPD patients with a history of sustained childhood abuse. Neuropsychopharmacology 28, 126–132. 10.1038/sj.npp.1300003 12496948

[B28] Roepke S. Ziegenhorn A. Kronsbein J. Merkl A. Bahri S. Lange J.. (2010). Incidence of polycystic ovaries and androgen serum levels in women with borderline personality disorder. J. Psychiatr. Res. 44, 847–852. 10.1016/j.jpsychires.2010.01.007 20149393

[B29] Sansone R. A. Sansone L. A. (2011). Gender patterns in borderline personality disorder. Innov. Clin. Neurosci. 8, 16. 21686143 PMC3115767

[B30] Sher L. (2013). Low testosterone levels may be associated with suicidal behavior in older men while high testosterone levels may be related to suicidal behavior in adolescents and young adults: a hypothesis. Int. J. Adol. Med. Health 25, 263–268. 10.1515/ijamh-2013-0060 23893672

[B31] Skabeikyte G. (2021). A systematic review of the factors associated with the course of borderline personality disorder symptoms in adolescence. Borderline Pers. Disorder Emot. Dysreg. 8, 2–14. 10.1186/s40479-021-00151-z 33866976 PMC8054370

[B32] Stepp S. D. Lazarus S. A. Byrd A. L. (2016). A systematic review of risk factors prospectively associated with borderline personality disorder: taking stock and moving forward. Personal. Disord. Theory Res. Treat. 7, 316. 10.1037/per0000186 27709988 PMC5055059

[B33] Streit F. Witt S. H. Awasthi S. Foo J. C. Jungkunz M. Frank J. (2022). Borderline personality disorder and the big five: molecular genetic analyses indicate shared genetic architecture with neuroticism and openness. Transl. Psychiatry 12, 153. 10.1038/s41398-022-01912-2 35411043 PMC9001677

[B34] Tan R. Y. M. Grigg J. Kulkarni J. (2018). Borderline personality disorder and polycystic ovary syndrome: a review of the literature. Aust. New Zeal. J. Psychiatry 52, 117–128. 10.1177/0004867417730650 28891300

[B35] Thomas N. Gurvich C. Hudaib A.-.R Gavrilidis E. Kulkarni J. (2019). Systematic review and meta-analysis of basal cortisol levels in borderline personality disorder compared to non-psychiatric controls. Psychoneuroendocrinology 102, 149–157. 10.1016/j.psyneuen.2018.12.009 30557762

[B36] Trisno R. Worsley R. Kulkarni J. (2016). Borderline personality disorder and polycystic ovary syndrome. Aust. Psychiatry 50, 385. 10.1177/0004867415615950 26553218

[B37] Winsper C. (2018). The aetiology of borderline personality disorder (BPD): contemporary theories and putative mechanisms. Curr. Opin. Psychol. 21, 105–110. 10.1016/j.copsyc.2017.10.005 29182951

[B38] Winsper C. Bilgin A. Thompson A. Marwaha S. Chanen A. M. Singh S. P.. (2020). The prevalence of personality disorders in the community: a global systematic review and meta-analysis. Br. J. Psychiatr. 216, 69–78. 10.1192/bjp.2019.166 31298170

[B39] Winsper C. Marwaha S. Lereya S. T. Thompson A. Eyden J. Singh S. P.. (2016). A systematic review of the neurobiological underpinnings of borderline personality disorder (BPD) in childhood and adolescence. Rev. Neurosci. 27, 827–847. 10.1515/revneuro-2016-0026 27518904

[B40] Woodbridge J. Reis S. Townsend M. L. Hobby L. Grenyer B. F. (2021). Searching in the dark: Shining a light on some predictors of non-response to psychotherapy for borderline personality disorder. PLoS ONE. 16, e0255055. 10.1371/journal.pone.0255055 34314461 PMC8315515

[B41] Zanarini M. C. Vujanovic A. A. Parachini E. A. Boulanger J. L. Frankenburg F. R. Hennen J.. (2003). A screening measure for BPD: the McLean screening instrument for borderline personality disorder (MSI-BPD). J. Pers. Disord. 17, 568–573. 10.1521/pedi.17.6.568.25355 14744082

[B42] Zilioli S. Ponzi D. Henry A. Maestripieri D. (2015). Testosterone, cortisol and empathy: evidence for the dual-hormone hypothesis. Adapt. Hum. Behav. Physiol. 1, 421–433. 10.1007/s40750-014-0017-x

